# Development of Hepatitis C Virus Genotyping by Real-Time PCR Based on the NS5B Region

**DOI:** 10.1371/journal.pone.0010150

**Published:** 2010-04-13

**Authors:** Sueli M. Nakatani, Carlos A. Santos, Irina N. Riediger, Marco A. Krieger, Cesar A. B. Duarte, Marco A. Lacerda, Alexander W. Biondo, Flair J. Carilho, Suzane K. Ono-Nita

**Affiliations:** 1 Laboratório Central do Estado (LACEN-PR), São José dos Pinhais, Paraná, Brazil; 2 Centro de Genomas, São Paulo, São Paulo, Brazil; 3 Instituto Carlos Chagas – Fundação Oswaldo Cruz (ICC-FioCruz), Curitiba, Paraná, Brazil; 4 Division of Gastroenterology and Hepatology, Indiana University, Indianapolis, Indiana, United States of America; 5 Department of Veterinary Medicine, Federal University of Paraná, Curitiba, Brazil; 6 Department of Pathobiology, University of Illinois, Urbana, Illinois, United States of America; 7 Department of Gastroenterology, School of Medicine, University of São Paulo (USP), São Paulo, Brazil; 8 Departamento de Biologia Celular, Universidade Federal do Paraná, Curitiba, Brazil; HelmholtzZentrum München, Germany

## Abstract

**Background:**

Hepatitis C virus (HCV) genotyping is the most significant predictor of the response to antiviral therapy. The aim of this study was to develop and evaluate a novel real-time PCR method for HCV genotyping based on the NS5B region.

**Methodology/Principal Findings:**

Two triplex reaction sets were designed, one to detect genotypes 1a, 1b and 3a; and another to detect genotypes 2a, 2b, and 2c. This approach had an overall sensitivity of 97.0%, detecting 295 of the 304 tested samples. All samples genotyped by real-time PCR had the same type that was assigned using LiPA version 1 (Line in Probe Assay). Although LiPA v. 1 was not able to subtype 68 of the 295 samples (23.0%) and rendered different subtype results from those assigned by real-time PCR for 12/295 samples (4.0%), NS5B sequencing and real-time PCR results agreed in all 146 tested cases. Analytical sensitivity of the real-time PCR assay was determined by end-point dilution of the 5000 IU/ml member of the OptiQuant HCV RNA panel. The lower limit of detection was estimated to be 125 IU/ml for genotype 3a, 250 IU/ml for genotypes 1b and 2b, and 500 IU/ml for genotype 1a.

**Conclusions/Significance:**

The total time required for performing this assay was two hours, compared to four hours required for LiPA v. 1 after PCR-amplification. Furthermore, the estimated reaction cost was nine times lower than that of available commercial methods in Brazil. Thus, we have developed an efficient, feasible, and affordable method for HCV genotype identification.

## Introduction

Hepatitis C virus (HCV) genotyping is reported to be the primary tool for assessing the course of infection and determining treatment duration [1]. Several molecular methods targeting different HCV genomic regions have been described for determining genotype [2], [3]. Most commercial methods target the 5′ untranslated region (5′UTR), which is the region of choice for qualitative and quantitative HCV RNA detection because of its high degree of conservation among different subtypes [4]. The 5′ UTR, however, does not allow sufficient discrimination between closely related subtypes within the same genotype. Subtyping is particularly useful for investigating infection outbreaks and for better understanding the epidemiological and virological features of HCV [5]. In addition, accurate subtyping may be important for future studies aiming to develop new drugs and to increase our understanding of how HCV becomes resistant to therapies [6].

Sequencing of an appropriate coding region, such as the nonstructural 5B region (NS5B), core, or E1 is the gold standard for discriminating HCV types and subtypes [7]. Despite of the usefulness of this approach, sequencing is a laborious technique and typically requires several sample handling steps. This increases turnaround time, thus impairing patient diagnosis and treatment.

Methods such as real-time PCR are specific and sensitive because they simultaneously use probes and primers to detect target sequences [8]. Moreover, this technique is performed on an automated platform without the need for post-PCR procedures, thus minimizing cross-contamination between samples and accelerating the analysis. The aim of this study was to develop, standardize and validate a method for identifying HCV genotypes by real time PCR based on the analysis of the HCV NS5B region.

## Results

**Figure 1:**
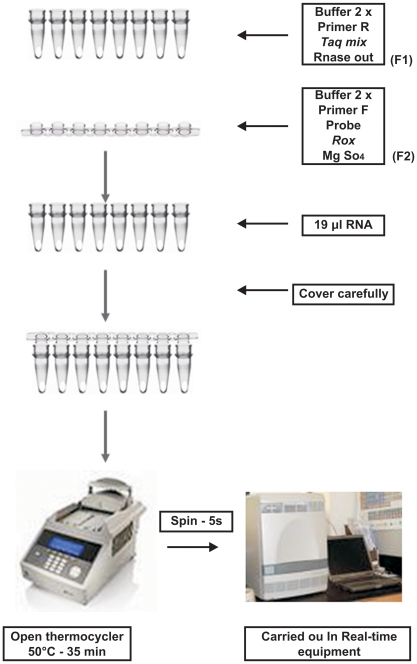
Schematic view of modified one-step real-time PCR. The reaction mix was prepared in two fractions, in tubes and caps.

We have designed and standardized a new real-time PCR-based method for genotyping HCV. This method consists of two triplex reactions targeting genotypes 1, 2 and 3. Both reverse transcription and amplification were carried out in the same reaction tube, as detailed in Figure 1.

### Real-Time PCR genotyping validation tests

#### Accuracy

Results obtained by real-time PCR genotyping and partial sequencing of the NS5B region showed 100% concordance in all 146 tested samples. No discrepancies were found using phylogenetic analysis or matching with Blast.

#### Reproducibility

Assays were performed for genotypes 1a, 1b, and 3a on three different days with 16 replicates (n = 48) of each genotype/sample. The total coefficient of variation of the cycle threshold (C_t_) was 2.67% for genotype 3a (mean C_t_, 27.49±0.73), 6.25% for genotype 1b (mean C_t_, 26.54±1.66), and 6.40% for genotype 1a (mean C_t_, 27.65±1.73).

#### Sensitivity

Analytical sensitivity was determined by analyzing the Optiquant® HCV RNA Quantification Panel (Acrometrix, Benicia, CA, USA). The 95% detection limit was measured at 250 IU/ml. Relative sensitivity based on patients infected with genotypes 1a, 2b, or 3a showed a 95% detection limit of 500 IU/ml for genotype 1a, 250 IU/ml for genotype 2b, and 125 IU/ml for genotype 3a.

#### Specificity

Based on 53 blood donor samples, when the two primer-probe sets were used for real-time PCR genotyping, no false-positive reactions were detected. Analytical specificity was assessed by evaluation of each genotype-specific primer-probe set with genotypes 4a and 5a. No false-positive results were obtained when those genotypes were tested with each genotype-specific primer-probe set. Based on 40 samples from patients with diseases unrelated to HCV, the two primer-probe sets were found to be adequately specific, not leading to false-positive results with common hepatitis viruses (HAV and HBV), dengue virus or HIV.

### *In vitro* mixed infections

After testing *in vitro* prepared mixtures containing different proportions of RNA of genotypes 1a and 3a, both genotypes were correctly detected in all the tested mixtures.

### Correlation between real-time PCR genotyping and LiPA

A total of 304 clinical samples were used to compare the real-time PCR genotyping with LiPA v. 1 (Table 1). Of those, nine samples could not be amplified and genotyped by real-time PCR, leading to 3% of false-negative results. Of the remaining 295 samples, genotyping by real-time PCR detected 100% (184/184) of the samples assigned as genotype 1 by LiPA v. 1. Mean C_t_ of real-time PCR genotyping for genotype 1a was 26.43 and 27.24 for genotype 1b. Real-time PCR genotyping also produced concordant results for all the 31 samples classified as genotype 2 by LiPA v. 1, with mean C_t_'s of 25.83. Moreover, real-time PCR was able to genotype 80/80 (100%) samples of genotype 3 with mean C_t_'s of 26.34. At the type level, a concordance of 100% was observed for the 295 samples when real-time PCR genotyping and LiPA v. 1 were compared. In contrast, when we compared the results of the two methods at the subtype level, the real-time PCR test was able to subtype the 68 samples non-subtyped by LiPA v. 1, as detailed in Table 1. Of those, 55 (80.9%) were sequenced and the results assigned by real-time PCR were confirmed.

**Table 1:** Comparison of the HCV genotyping results obtained with LiPA version 1 (5′UTR) and Real-Time PCR genotyping (NS5B).

LiPA (5′UTR)	Real-time PCR genotyping (NS5B)							
	1a	1b	2a	2b	2c	3a	Negative	Total
1	35**^35^**	7					1	43^35^
1a	47	5**^3^**						52^3^
1b	7**^5^**	75					3	85^5^
1a/1b	4**^4^**	4**^3^**						8^7^
2			1	14**^11^**			4	19^11^
2a/2c					3**^2^**		1	4^2^
2b				13				13
3a						80		80
	95^44^	89^6^	1	27^11^	3^2^	80	9	Total

Superscript data represent the number of samples sequenced and confirmed by NS5B partial sequencing.

Subtype classification discrepancies were found in 4.0% (12/295) of the samples. The genotyping concordance between real-time PCR and LiPA v. 1 was moderate (κ coefficient = 0.1111, *P* = 0,2218) when discrepant and non-subtyped results were taken into account. Ten of the 12 (83.3%) samples with discrepant subtyping results were sequenced and the results obtained from real-time PCR genotyping were confirmed.

### Estimation of costs

When the costs related to the real-time PCR-based genotyping test were compared to those of LiPA v. 1, a calculated nine-time decrease of unitary test cost estimation was observed.

## Discussion

In this study we described the development of a novel HCV genotyping method based on real-time PCR that involves two one-step triplex reactions. One reaction detects genotypes 1a, 1b, and 3a; while the other detects genotypes 2a, 2b, and 2c. The present method targets the NS5B region, which may be useful for epidemiological studies as well as for correlating patient response to current and new drugs as a function of infection with different HCV types and subtypes. Accuracy, precision, analytical and relative sensitivity and specificity were investigated in order to validate the method. Performance was evaluated by comparing the genotyping results obtained from real-time PCR with those generated by LiPA v. 1 and partial NS5B sequencing.

Some studies have reported real-time PCR methods for HCV genotyping. Two studies have described a single-step, real-time, reverse transcription-PCR (RT-PCR) reaction using a genotype-specific hydrolysis probe [9],[10], LightCycler probes [11],[12] or SYBR Green detection [13]. All these methods focus on the 5′ UTR. More recently, a real-time PCR-based method has become commercially available (Abbott HCV Genotype ASR). The assay design of this kit is such that genotypes 1a and 1b are typed by amplification targeting in the NS5B region. Other genotypes such as 2, 3, 4, 5, and 6 are detected through the 5′ UTR. Cook [14] compared this commercial method with a restriction fragment polymorphism (RLFP) method and discrepant samples were sequenced. Results showed the analytical sensitivity to be poor for genotypes 1a and 1b, even when these were present at >1500 UI/ml. In contrast, our method shows a lower limit of detection in the genotype-specific reactions ranging from 125 IU/ml for genotype 3a, to 500 IU/ml for genotype 1a. The current genotyping method presented an overall detection rate of 295/304 (97.0%), which is similar to previously reported PCR-based methods [3].

It has been demonstrated that there is no difference in the relative efficiency of one-step and two-step systems when the expression rate of the target gene is high [3]. When the rate of expression is low, however, detection occurred five PCR cycles earlier in the one-step method than in the two-step method. Based on these findings, we sought to standardize our method as a one-step system instead of a two-step one. The next step was to choose an enzyme that could work at higher temperatures in the reverse transcription reaction. Different studies show that the choice of enzyme is a key determinant of reaction efficiency [15],[16]. In this study, we used a one-step kit with Superscript™ III, a modified version of MMLV-RT that is stable at 45–60°C; the use of these elevated temperatures reduces the formation of secondary structures and the RNase H activity associated with the reverse transcriptase [17].

Studies have shown that addition of forward primers after the reverse transcription reaction and before activation of *Taq* DNA polymerase prevents the formation of primer-dimers and results in more efficient amplification [16]. We therefore tested a reaction modification in which the reagents were added separately. Some of the reagents were placed in the reaction tube and the remaining reagents, including the forward primer, were placed in the tube caps and spun into the reaction tubes after the completion of the reverse transcription step. The modified one-step method showed earlier amplification (5.6 C_t_'s on average) than the original one-step method. In addition, by placing the forward primer in the optical cap, the reaction could be carried out without opening the tube, thereby avoiding potential contamination between samples and enabling the reaction to be performed in a single step.

The cost-benefit of a triplex reaction was also considered when setting up the assay. Therefore, a single primer set was designed to amplify genotypes 1a, 1b and 3a. Probes labeled with the fluorophores FAM, VIC and NED, which use different optical reading channels, were used to detect PCR products. An equivalent triplex reaction was set to detect subtypes 2a, 2b, and 2c, and the melting temperature was calculated to be 60°C for primers and 70°C for probes.

To avoid the inclusion of polymorphic positions within the NS5B region, shorter oligonucleotides were designed into the probes of genotypes 1a, 1b, and 3a. Consequently, the estimated melting temperature was 66°C for probes and 56°C for primers, and an additional cycling step of 50°C was added to the real-time PCR. The above types and subtypes were selected because they account for 99% of the HCV-infected population in Brazil [18]. In contrast, genotypes 4 and 5 account for 0.3% of the Brazilian cases [19] and genotype 6 has never been described in Brazil [20].

A recent study of real-time PCR genotyping has proposed extracting RNA from a plasma volume of 1,000 µl eluting it into 80 µl, which corresponds to a 12.5-fold concentration [14]. In the present study, because of the limited amount of plasma available to us, we extracted RNA from 200 µl of plasma and eluted it into 60 µl, corresponding to a 3.3-fold concentration. The lower concentration was compensated by adding 19 µl of RNA to the reaction.

Validation tests confirmed the specificity of the present method, both in relation to different genotypes and to the potential presence of a mixed viral population. Results showed that this method can detect multiple genotypes at all dilutions tested, even when the minor genotype is present at 10-fold lower levels than the major genotype. However, more samples must be analyzed in order to determine the true prevalence of mixed infections in our population.

When HCV genotyping results generated by real-time PCR and LiPA v. 1 were compared, 100% concordance was found at the type level. These results are similar to those reported by previous study involving 357 samples from blood donors in France, which demonstrated 100% concordance between 5′ UTR and NS5B sequence analysis when classifying types [4]. However, genotype identification based on the 5′ UTR may pose a problem in some geographical regions, because some genotype 6 variants found in Southeast Asia have identical 5′ UTR sequences to those of genotypes 1a or 1b [3].

At the subtype level, however, LiPA v. 1 was unable to subtype 68 of the 295 samples (23.0%), all of which were correctly typed by real-time PCR as confirmed by sequencing. A misclassification frequency of 4.0% (12/295) was observed when comparing the genotyping results of real-time PCR and LiPA v. 1. This lack of agreement includes cases in which the two methods gave different subtype results. In this study, LiPA v. 1 misclassified seven samples of genotype 1a as genotype 1b, yielding a misclassification rate of 12.9% for subtype 1a. This finding is consistent with other study that have found that amplifying the 5′ UTR overestimates the frequency of subtype 1b by 20% at the expense of subtype 1a [21]. Furthermore, LiPA v. 1 was unable to assign the subtype for eight samples which were typed as 1a/1b. Using real-time PCR genotyping, four of these samples were classified as 1b and the remaining four as genotype 1a.

The real-time PCR genotyping method described herein showed high accuracy at the subtype level but failed to amplify 3% of the samples. These results show that targeting a polymorphic region potentially impairs amplification efficiency since potential mutations may reduce the affinity between the target region and the PCR primers and probes.

In addition to its robustness, the real-time PCR method costs 9 times less than the commercial methods currently available in Brazil, when the costs of reagents and laboratory material are taken into account. Another positive feature of our proposed method is its fewer handling steps and its faster turnaround time. Since in Brazil the prevalence of genotypes 4 and 5 is low while genotype 6 has never been described, those types were not taken into account when designing this method. However, the same detection platform described herein can be used to design complementary assays to detect genotypes 4, 5 and 6.

This method may provide more accurate correlation between clinical outcomes and different genotypes and their subtypes, as well as improved understanding of the role of genomic variability on HCV infectiousness and on its demographic pattern of infection.

## Materials and Methods

### Sample selection

Ethylenediaminetetraacetic acid (EDTA) plasma was prospectively collected between January 2005 and February 2008 from 304 patients chronically infected with HCV who were admitted to the Paraná State Reference Laboratory of Health (LACEN-PR) and the Clinics Hospital at the University of São Paulo. Plasma was stored at −70°C until use. Sample selection was based on HCV genotype as had been previously assigned using the Versant™ HCV genotype Assay-LiPA (version I; Siemens Medical Solutions, Diagnostics Division, Fernwald, Germany). The study protocol was approved by the Ethics Committee of University of São Paulo (CAAE –2546.0.015.000-05). The requirement for written informed consent was waived by the IRB because the samples used were coded upon collection and storage. Besides, the study did not present any risks for the patients and involved no procedures for which written consent is normally required outside of the research context.

### RNA extraction

A total of 200 µl of EDTA plasma was extracted using the automated platform Nuclisens easyMAG (bioMérieux, Boxtel, Netherlands) according to the manufacturer's instructions. The RNA was eluted in 60 µl of elution buffer. The total RNA was quantified using the Quant-iT™ RNA assay kit (Invitrogen, Carlsbad, CA, USA). The observed mean quantity was 30,6 ng.

### Real-time PCR genotyping

Primers were designed to anneal to conserved sites within the NS5B region and probes were designed to target the internal polymorphic positions in this genomic region. Oligonucleotide design was based on the analysis of data provided by the Los Alamos National Laboratory (http://hcv.lanl.gov/components/hcv-db/GET_ALIGNMENTS/get_alignment.comp).

One primer pair and three different probes labeled with different fluorophores were designed to amplify and detect genotypes 1a, 1b, and 3a. An equivalent strategy was used to detect genotypes 2a, 2b, and 2c. Sequences, locations and specificities of primers and probes are shown in Table 2. Oligonucleotide melting temperatures were calculated using Primer Express (Applied Biosystems, Foster, CA, USA).

**Table 2:** Primers and Probes used in Real-Time PCR genotyping of NS5B region.

Primer	Genotype	Sequences (5′-3′)	Product	Position^1^
F56_1–3	1 and 3	5′-CACACTCCAGTY^C/T^AAY^C/T^TCCTGG-3′	241 bp	1204–1224
R56 1–3		5′-CW^A/T^M^A/C^CTGGAGAGTAACTGTGGAG-3′		1444–1423
GEN2FSN	2	5′-GAC ACT CCC CTG TCA ATT CW^A/T^T GG-3′	120 bp	1202–1224
GEN2RSN		5′-TG GTY^C/T^ CAG AGT GTC Y^C/T^TG GGC-3′		1321–1313

Probe	Genotype	Fluorophore	Sequences	Quencher	Position^1^
1a66	1a	6-FAM	5′-CAGCTTGAACAGGC-3′	NFQ-MGB	1312–1325
1b266	1b	VIC	5′-CAACTTGAAAAAGC-3′	NFQ-MGB	1312–1325
3a66	3a	NED	5′-AGATACTTGATCGACCC-3′	NFQ-MGB	1312–1325
2a	2a	NED	5′-TTCTCCATTCTCAT-3′	NFQ-MGB	1287–1300
2b	2b	6-FAM	5′-TTCTCCATACTATT-3′	NFQ-MGB	1287–1300
2c	2c	VIC	5′-TTCTCCGTCTTGAT-3′	NFQ-MGB	1287–1300

1 1a_.H77.NC_004102–reference sequence used to calculate positions of NS5B region.

### Modified one-step real-time PCR

Reactions were performed in a final volume of 50 µl using the Superscript™ III Platinum® one-step quantitative RT-PCR system (Invitrogen). The reaction mix was prepared in two fractions. The first fraction (F1) consisted of 18.5 µl of 2X reaction mix (6 mM MgSO_4_, and 0.4 mM of each dNTP), 1.0 µl of *Taq* mix (Superscript™III RT, Platinum *Taq* Mix), 200 nM of reverse primer (R56_1–3 or gen2RSN), and 0.5 µl of RNase Out (Invitrogen). Twenty-one microliters of F1 were added to optical tubes. The second reaction mix fraction (F2) consisted of 4.4 µl of 2X reaction mix, 200 nM of forward primer (F56_1–3 or gen2FSN), 1.5 µl of 50 mM MgSO_4_, 0.1 µl of ROX (25 µM), and 200 nM of each probe (against either genotypes 1a, 1b, and 3a; or against genotypes 2a, 2b, and 2c). A final F2 volume of 10 µl was transferred to the caps of the optical tubes. After the addition of 19 µl of RNA, the optical tubes (containing F1) were carefully covered with the caps (containing F2). Reverse transcription was carried out in a GeneAmp PCR System 9700 (Applied Biosystems) for 35 min at 50°C with the thermocycler cover open. Afterwards, tubes were briefly centrifuged and real-time PCR was carried out in a 7500 Real-Time PCR System (Applied Biosystems, Foster, CA, USA) using the following cycling parameters: 2 min at 50°C; 10 min at 95°C; 40 cycles of 15 s at 95°C, 30 s at 50°C; and 1 min at 60°C (for set 1a, 1b, and 3a) or 15 s at 95°C and 1 min at 60°C (for set 2a, 2b, and 2c).

### Real-time PCR performance tests

Real-time PCR precision was assessed by testing three randomly selected clinical samples. Sixteen replicates of each sample were analyzed on three different days (n = 48).

Analytical sensitivity was investigated using the genotype 1b OptiQuant® HCV RNA Quantification Panel (Acrometrix, Benicia, CA, USA). The lower limit of detection was determined as the lowest concentration needed to provide genotype identification with a hit rate of at least 95% of 24 replicates. The panel member containing 5,000 IU/ml was diluted in negative human plasma to 1,000, 500, 250, and 125 IU/ml. Relative sensitivity was determined for genotypes 1a, 2b, and 3a by analyzing 8 replicates of clinical specimens. Based on the HCV viral load assigned by the Cobas Amplicor® HCV Test (version 2.0; Roche, Branchburg, NJ, USA), each specimen was diluted to 500, 250, and 125 IU/ml. Eight replicates of each dilution were analyzed.

Specificity was assessed by testing 53 samples from healthy, HCV-negative blood donors provided by the Blood Bank from Clinics Hospital of University of São Paulo.

To assess analytical specificity and possible false-positive detection by the genotype-specific primer-probe sets, we tested specimens infected with Hepatitis B Virus (n = 10), Hepatitis A Virus (n = 10), HIV (n = 10), and dengue virus (n = 10), which belongs to the same family (Flaviviridae) as HCV. HIV-infected samples were chosen because of the presumed high incidence of HIV-HCV co-infection in the population studied. Those samples tested negative for HCV RNA in the Amplicor® HCV Test (version 2.0; Roche). We also included samples infected with genotypes 4a (4 samples) and 5a (1 sample) to test for cross-amplification and detection with genotypes 1, 2 and 3.

### *In vitro* mixed infection

To determine whether the presence of hypothetical mixed infections interfered with the specificity of the assay, we artificially mixed different proportions of HCV RNA from different genotypes. One sample of genotype 1a (550,000 IU/ml) and a sample of genotype 3a (559,000 IU/ml) were mixed in proportions of 1∶1, 1∶5, and 1∶10 for 1a and 3a and vice-versa.

### One-step real-time PCR

Reactions were performed with the same concentrations of reagents and cycling parameters described above, with a final reaction volume of 50 µl, except that the reaction mix was prepared in a single fraction rather than two.

### Partial sequencing of HCV NS5B region

Partial NSB5 sequencing was performed for 146 of the 304 specimens to verify the accuracy of the HCV genotypes determined by the triplex HCV genotype PCR reactions. Reverse transcription was performed essentially as previously described [22] using 300 U of Moloney Murine Leukemia Virus RT (MMLV-RT; Invitrogen) and 7.5 ng/µl of random primer (cat. # 48190-011; Invitrogen).

Primers NS5B2F (5′-TTCACGGAGGCTATGACY^C/T^AGG-3′) and NS5B-2R (5′-CGGGCATGM^A/C^GACAS^G/C^GCTGTGA-3′), located between the positions 1683 and 1704 of the NS5B region, were used for partial sequencing of the NS5B region generating an expected product of 688 bp. Each PCR reaction contained buffer [10X concentrations: 200 mM Tris-HCl (pH 8.4), 500 mM KCl], 1.5 mM MgCl_2_, 200 nM of each dNTP, 1 µM of each primer, 2.5 IU of High Fidelity *Taq* DNA polymerase (Invitrogen), and 5 µl of cDNA to be tested. The final volume was adjusted to 50 µl using ultrapure water. PCR was carried out using a GeneAmp PCR System 9700 (Applied Biosystems). The following amplification conditions were used: 5 min at 94°C; 35 cycles at 95°C for 30 s, 60.5°C for 1 min for primers NS5B2F/NS5B2R or 56°C for 45 s for primers F56_1–3/R56_1–3 (Table 2), 72°C for 1 min; 10 min at 72°C and a final hold at 10°C.

Sequencing reactions were performed in both directions using the Big Dye terminator technology (version 3.2; Applied Biosystems) and products were detected with an ABI 3130 Genetic Analyzer (Applied Biosystems). Sequencing analysis was performed using SeqScape (Applied Biosystems), and genotypes were assigned by matching using Blast (http://blast.ncbi.nlm.nih.gov/Blast.cgi).

The generated sequences were submitted to GenBank and can be retrieved under accession numbers FJ159697 to FJ159849.

### Phylogenetic analysis

An internal fragment of 216 nucleotides within the PCR products generated for sequencing (positions 1223–1438 in the NS5B region according to the reference sequence H77, GenBank accession number NC004102) was used for phylogenetic analysis. Nucleotide distances were computed on MEGA version 4.0 [23] by the ρ-distance algorithm and phylogenetic trees were inferred using the neighbor joining method. Robustness of tree branches was tested by bootstrap analysis (1000 replicates).

### Statistical analysis

Consistency between real time PCR and LiPA was assessed using the κ coefficient, with κ values between 0.61 and 0.80 taken to indicate good concordance [24], when both methods classified specimens into the same categories (genotypes or subtypes). Statistical analysis was performed using Program R for Windows (version 2.7.0). Concord software was used to calculate agreement between techniques. A *P* value of less than 0.05 was considered significant.

### Unitary test cost estimation

Estimation of unitary test costs was based on the currently available commercial reagent prices in Brazil at the time the study was conducted. Equipment maintenance, human resources and other indirect costs were not taken into account for comparison and calculation.

To estimate the unitary cost of the real-time PCR test, costs of NucliSens EasyMAG extraction kit (bioMerieux), Superscript™ III Platinum One-Step qRT-PCR kit for 500 reactions (Invitrogen), TaqMan probes (synthesized by Applied Biosystems), primer sets (synthesized by Invitrogen), RNase Out (Invitrogen), and optical tubes and caps (Applied Biosystems) were taken for comparison. Assessment of the final costs related to LiPA v.1 included the Amplicor® Hepatitis C Vírus–HVC–Test, version 2.0 (Roche) and the VERSANT™ HCV Genotype Assay-LiPA v.1 (Siemens).
