# Correction: Development of Hepatitis C Virus Genotyping by Real-Time PCR Based on the NS5B Region

**DOI:** 10.1371/annotation/33b802f2-0973-4bc8-ab13-1dbf78e3b13d

**Published:** 2010-04-28

**Authors:** Sueli M. Nakatani, Carlos A. Santos, Irina N. Riediger, Marco A. Krieger, Cesar A. B. Duarte, Marco A. Lacerda, Alexander W. Biondo, Flair J. Carrilho, Suzane K. Ono-Nita

The eighth author's name is incorrect. The correct name is: Flair J. Carrilho.

